# 4′-(4-Chloro­phen­yl)-3′-(4-meth­oxy­phen­yl)-3,4-dihydro-1*H*,4′*H*-spiro­[acridine-2,5′-isoxazol]-1-one

**DOI:** 10.1107/S1600536812042523

**Published:** 2012-10-20

**Authors:** Ponmudisettu Narayanan, Shanmugavel Uma Maheswari, Krishnan Sethusankar

**Affiliations:** a Department of Physics, RKM Vivekananda College (Autonomous), Chennai 600 004, India; b Department of Chemistry, School of Organic Chemistry, Madurai Kamaraj University, Madurai 625 021, India

## Abstract

In the title compound, C_28_H_21_ClN_2_O_3_, the quinoline ring system is essentially planar with a maximum deviation of 0.0436 (17) Å. The isoxazole and cyclo­hexane rings adopt envelope conformations. The isoxazole ring is almost orthogonal to both the quinoline ring system and the cyclo­hexane ring, making dihedral angles of 85.75 (8) and 81.46 (9) °, respectively. The O atom deviates signifigantly from the six-membered carbocyclic ring by 0.3947 (16) Å. In the crystal, mol­ecules are linked into inversion dimers *via* pairs of C—H⋯O inter­actions, resulting in *R* ^2^_2_(24) ring motifs.

## Related literature

For the uses and biological importance of acridines, see: Asthana *et al.* (1991 ▶); Di Giorgio *et al.* (2005 ▶); Talacki *et al.* (1974 ▶). For related structures, see: Sridharan *et al.* (2009 ▶); Trzybiński *et al.* (2010 ▶). For graph-set notation, see: Bernstein *et al.* (1995 ▶). 
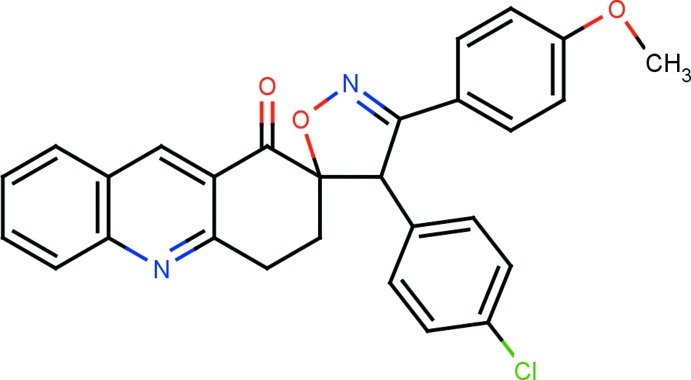


## Experimental

### 

#### Crystal data

C_28_H_21_ClN_2_O_3_
*M* *_r_* = 468.92
Monoclinic, 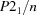

*a* = 12.1626 (4) Å
*b* = 16.2747 (6) Å
*c* = 12.1960 (4) Å
β = 107.704 (2)°
*V* = 2299.78 (14) Å^3^
*Z* = 4
Mo *K*α radiation
μ = 0.20 mm^−1^
*T* = 293 K
0.35 × 0.30 × 0.25 mm

#### Data collection

Bruker Kappa APEXII CCD diffractometer
Absorption correction: multi-scan (*SADABS*; Bruker, 2008 ▶) *T*_min_ = 0.932, *T*_max_ = 0.951
21438 measured reflections
4288 independent reflections
3335 reflections with *I* > 2σ(*I*)
*R*_int_ = 0.022

#### Refinement

*R*[*F* ^2^ > 2σ(*F* ^2^)] = 0.037
*wR*(*F* ^2^) = 0.108
*S* = 1.04
4288 reflections
308 parameters
H-atom parameters constrained
Δρ_max_ = 0.16 e Å^−3^
Δρ_min_ = −0.32 e Å^−3^

### 

Data collection: *APEX2* (Bruker, 2008 ▶); cell refinement: *SAINT* (Bruker, 2008 ▶); data reduction: *SAINT*; program(s) used to solve structure: *SHELXS97* (Sheldrick, 2008 ▶); program(s) used to refine structure: *SHELXL97* (Sheldrick, 2008 ▶); molecular graphics: *ORTEP-3* (Farrugia, 1997 ▶); software used to prepare material for publication: *SHELXL97* and *PLATON* (Spek, 2009 ▶).

## Supplementary Material

Crystal structure: contains datablock(s) global, I. DOI: 10.1107/S1600536812042523/pv2592sup1.cif

Structure factors: contains datablock(s) I. DOI: 10.1107/S1600536812042523/pv2592Isup2.hkl

Supplementary material file. DOI: 10.1107/S1600536812042523/pv2592Isup3.cml

Additional supplementary materials:  crystallographic information; 3D view; checkCIF report

## Figures and Tables

**Table 1:** Hydrogen-bond geometry (Å, °)

*D*—H⋯*A*	*D*—H	H⋯*A*	*D*⋯*A*	*D*—H⋯*A*
C22—H22*B*⋯O1^i^	0.96	2.54	3.351 (2)	142

Symmetry code: (i) 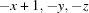.

**Fig. 1.**
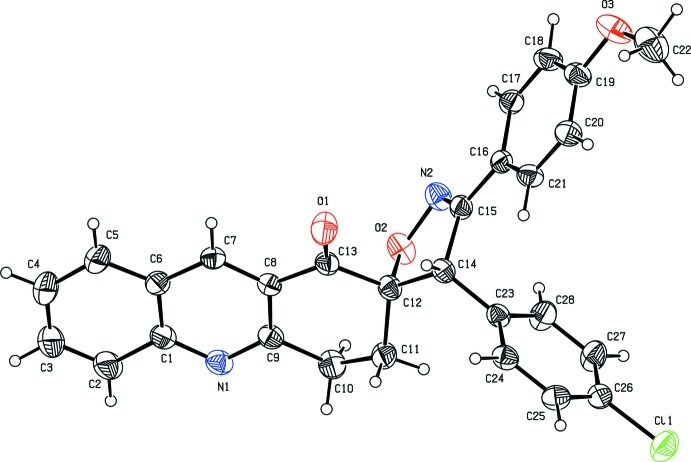
The molecular structure of the title compound showing intramolecular hydrogen bond, which generates S(5) ring motif with the atom numbering scheme. The displacement ellipsoids are drawn at 30% probability level.

**Fig. 2.**
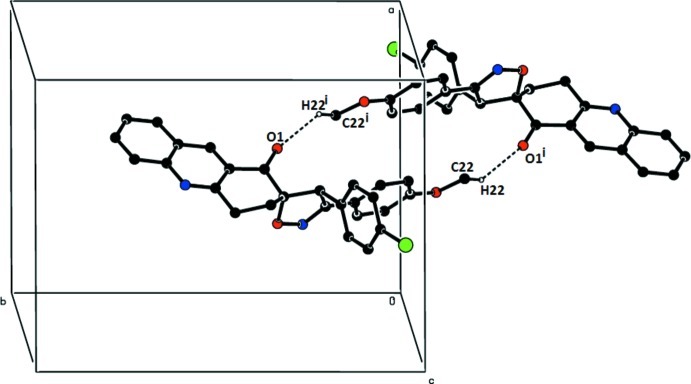
The crystal packing of the title compound, viewed down a-axis, showing C—H···O hydrogen bonds. The hydrogen atoms not involved in the hydrogen bonding have been excluded for clarity.

## supplementary crystallographic information

### Comment

Acridine derivatives are biologically important compounds, which are found to possess mutagenic, antitumour (Talacki *et al.*, 1974), antibacterial, antiamoebic, hypersensitive, antiinflammatory and antiimplantation (Asthana *et al.*, 1991) activities. Also, they have been shown to exert toxicity towards plasmodium, trypanosoma and leishmania parasites (Di Giorgio *et al.*, 2005). Against this backround, X-ray study of the title compound has been carried out to study its structural aspects.

The title compound (Fig. 1), comprises an acridinone ring system, an isoxazole ring attached to a chlorophenyl and a methoxyphenyl rings. The quinoline ring system forms a dihedral angle of 88.06 (7) ° with the chlorophenyl ring (C23–C28), which shows that they are almost orthogonal to each other. The methoxy phenyl ring (C16–C21) forms a dihedral angle of 76.26 (7) ° with the quinoline ring system.

The cyclohexane ring (C8–C13) adopts a C11-*envelope* conformation with C11 0.3024 (19) Å out of the mean-plane formed by the remaining ring atoms. The cyclohexane ring forms a dihedral angle of 83.02 (8) ° with the chlorophenyl ring (C23–C28), which shows that they are almost perpendicular to each other. The oxygen atom (O1) significantly deviates from the cyclohexane by -0.3947 (16) Å. The methoxy phenyl ring (C16–C21) forms an interplanar angle of 71.43 (9) ° with the cyclohexane ring.

The isoxazole ring (N2/O2/C12/C14/C15) adopts a C12-*envelope* conformation with C12 0.1417 (17) Å out of the mean-plane formed by the remaining ring atoms. The isoxazole ring forms a dihedral angle of 85.75 (7) ° and 81.46 (9) ° with the quinoline bicyclic ring system and the cyclohexane ring, respectively. The title compound exibits structural similarities with already reported related structures (Sridharan *et al.*, 2009; Trzybiński *et al.*, 2010).

In the crystal, molecules are linked *via* C22—H22···O1^i^ intermolecular interactions resulting in *R*^2^_2_(24) graph-set ring motifs (Tab. 1 & Fig. 2).

### Experimental

A mixture of 3,4-dihydroacridin-1(2*H*)-one(200 mg, 1 mmol), 4-chloro benzaldehyde (168 mg, 1.2 mmol) and KOH (84 mg, 1.5 mmol) in dimethoxy ethane (DME) (3 ml) was stirred at ambient temperature for 30 min. Then, *N*-hydroxy-4-ethoxybenzimi doyl chloride (278 mg, 1.5 mmol) was added subsequently to the reaction mixture and stirred at room temperature for 10–12 h. The progress of the reaction was monitored by thin-layer chromatography with petroleum ether-ethyl acetate (4:1 *v*/*v*) mixture as eluent. After completion of the reaction, the reaction mixture was extracted with ethyl acetate (2x20 ml), washed with water (2x10 ml), dried over anhydrous Na_2_SO_4_ and concentrated under reduced pressure. The residue thus obtained was recrystallized from diethyl-ether to afford the title compound as off white solid. Single crystals suitable for X-ray diffraction were prepared by slow evaporation of a solution of the title compound in ethanol at room temperature (Yield = 88%; m.p. = 463–465 K).

### Refinement

The positions of the hydrogen atoms were localized from the difference electron density maps and the distances were geometrically constrained using a riding model, with C—H = 0.93, 0.96, 0.97 and 0.98 Å, for aryl, methyl, methylene and methine H-atoms, respectively; the rotation angles for methyl groups were optimized by least squares. The *U*_iso_(H) were allowed at 1.2*U*_eq_(C).

### Crystal data

**Table:**

C_28_H_21_ClN_2_O_3_	*F*(000) = 976
*M**_r_* = 468.92	*D*_x_ = 1.354 Mg m^−^^3^
Monoclinic, *P*2_1_/*n*	Mo *K*α radiation, λ = 0.71073 Å
Hall symbol: -P 2yn	Cell parameters from 4288 reflections
*a* = 12.1626 (4) Å	θ = 2.1–25.5°
*b* = 16.2747 (6) Å	µ = 0.20 mm^−^^1^
*c* = 12.1960 (4) Å	*T* = 293 K
β = 107.704 (2)°	Block, colourless
*V* = 2299.78 (14) Å^3^	0.35 × 0.30 × 0.25 mm
*Z* = 4	

### Data collection

**Table:**

Bruker Kappa APEXII CCD diffractometer	4288 independent reflections
Radiation source: fine-focus sealed tube	3335 reflections with *I* > 2σ(*I*)
Graphite monochromator	*R*_int_ = 0.022
ω & φ scans	θ_max_ = 25.5°, θ_min_ = 2.1°
Absorption correction: multi-scan (*SADABS*; Bruker, 2008)	*h* = −14→14
*T*_min_ = 0.932, *T*_max_ = 0.951	*k* = −19→12
21438 measured reflections	*l* = −14→14

### Refinement

**Table:**

Refinement on *F*^2^	Primary atom site location: structure-invariant direct methods
Least-squares matrix: full	Secondary atom site location: difference Fourier map
*R*[*F*^2^ > 2σ(*F*^2^)] = 0.037	Hydrogen site location: inferred from neighbouring sites
*wR*(*F*^2^) = 0.108	H-atom parameters constrained
*S* = 1.04	*w* = 1/[σ^2^(*F*_o_^2^) + (0.050*P*)^2^ + 0.5964*P*] where *P* = (*F*_o_^2^ + 2*F*_c_^2^)/3
4288 reflections	(Δ/σ)_max_ < 0.001
308 parameters	Δρ_max_ = 0.16 e Å^−^^3^
0 restraints	Δρ_min_ = −0.32 e Å^−^^3^

### Special details

**Table:**

Geometry. All e.s.d.'s (except the e.s.d. in the dihedral angle between two l.s. planes) are estimated using the full covariance matrix. The cell e.s.d.'s are taken into account individually in the estimation of e.s.d.'s in distances, angles and torsion angles; correlations between e.s.d.'s in cell parameters are only used when they are defined by crystal symmetry. An approximate (isotropic) treatment of cell e.s.d.'s is used for estimating e.s.d.'s involving l.s. planes.
Refinement. Refinement of *F*^2^ against ALL reflections. The weighted *R*-factor *wR* and goodness of fit *S* are based on *F*^2^, conventional *R*-factors *R* are based on *F*, with *F* set to zero for negative *F*^2^. The threshold expression of *F*^2^ > σ(*F*^2^) is used only for calculating *R*-factors(gt) *etc*. and is not relevant to the choice of reflections for refinement. *R*-factors based on *F*^2^ are statistically about twice as large as those based on *F*, and *R*- factors based on ALL data will be even larger.

### Fractional atomic coordinates and isotropic or equivalent isotropic displacement parameters (Å^2^)

**Table:**

	*x*	*y*	*z*	*U*_iso_*/*U*_eq_	
C1	0.51626 (16)	0.61689 (10)	0.26429 (14)	0.0516 (4)	
C2	0.5366 (2)	0.70100 (12)	0.29072 (18)	0.0739 (6)	
H2	0.5095	0.7251	0.3467	0.089*	
C3	0.5955 (2)	0.74654 (13)	0.2347 (2)	0.0889 (8)	
H3	0.6092	0.8018	0.2531	0.107*	
C4	0.6362 (2)	0.71204 (13)	0.1497 (2)	0.0869 (7)	
H4	0.6762	0.7446	0.1120	0.104*	
C5	0.61798 (19)	0.63173 (12)	0.12133 (17)	0.0660 (5)	
H5	0.6451	0.6094	0.0642	0.079*	
C6	0.55787 (15)	0.58187 (10)	0.17851 (14)	0.0482 (4)	
C7	0.54119 (14)	0.49764 (10)	0.15899 (13)	0.0446 (4)	
H7	0.5686	0.4720	0.1043	0.054*	
C8	0.48489 (13)	0.45288 (9)	0.21986 (13)	0.0423 (4)	
C9	0.44077 (14)	0.49391 (10)	0.30074 (14)	0.0475 (4)	
C10	0.37302 (18)	0.44738 (11)	0.36349 (18)	0.0627 (5)	
H10A	0.2916	0.4526	0.3218	0.075*	
H10B	0.3857	0.4719	0.4388	0.075*	
C11	0.40392 (17)	0.35664 (11)	0.37858 (16)	0.0567 (5)	
H11A	0.4803	0.3508	0.4333	0.068*	
H11B	0.3497	0.3288	0.4100	0.068*	
C12	0.40180 (14)	0.31634 (10)	0.26607 (14)	0.0467 (4)	
C13	0.48103 (15)	0.36219 (10)	0.21037 (14)	0.0468 (4)	
C14	0.42268 (14)	0.22334 (10)	0.26635 (14)	0.0448 (4)	
H14	0.5013	0.2130	0.2640	0.054*	
C15	0.33752 (14)	0.20169 (10)	0.15118 (14)	0.0449 (4)	
C16	0.33480 (13)	0.12310 (10)	0.09231 (13)	0.0435 (4)	
C17	0.26043 (14)	0.10992 (11)	−0.01813 (14)	0.0501 (4)	
H17	0.2099	0.1513	−0.0549	0.060*	
C18	0.26084 (16)	0.03676 (12)	−0.07315 (16)	0.0584 (5)	
H18	0.2120	0.0296	−0.1477	0.070*	
C19	0.33278 (15)	−0.02670 (11)	−0.01948 (15)	0.0536 (4)	
C20	0.40733 (16)	−0.01496 (11)	0.08936 (15)	0.0540 (4)	
H20	0.4568	−0.0569	0.1262	0.065*	
C21	0.40808 (15)	0.05969 (10)	0.14348 (14)	0.0506 (4)	
H21	0.4595	0.0675	0.2167	0.061*	
C22	0.3844 (2)	−0.16692 (15)	−0.0262 (2)	0.0926 (8)	
H22A	0.4657	−0.1559	−0.0048	0.139*	
H22B	0.3664	−0.2130	−0.0777	0.139*	
H22C	0.3628	−0.1792	0.0415	0.139*	
C23	0.40203 (13)	0.17522 (10)	0.36436 (14)	0.0443 (4)	
C24	0.49351 (14)	0.14896 (10)	0.45562 (14)	0.0499 (4)	
H24	0.5685	0.1620	0.4575	0.060*	
C25	0.47519 (15)	0.10362 (11)	0.54396 (15)	0.0548 (4)	
H25	0.5374	0.0858	0.6047	0.066*	
C26	0.36501 (15)	0.08517 (10)	0.54149 (15)	0.0516 (4)	
C27	0.27278 (16)	0.11154 (13)	0.45300 (17)	0.0649 (5)	
H27	0.1979	0.0993	0.4525	0.078*	
C28	0.29163 (16)	0.15627 (13)	0.36468 (16)	0.0629 (5)	
H28	0.2290	0.1740	0.3043	0.075*	
N1	0.45730 (13)	0.57296 (9)	0.32294 (13)	0.0543 (4)	
N2	0.26411 (13)	0.25830 (9)	0.10843 (13)	0.0567 (4)	
O1	0.53811 (14)	0.32611 (8)	0.16053 (13)	0.0750 (4)	
O2	0.28725 (10)	0.32714 (7)	0.18368 (12)	0.0614 (3)	
O3	0.32257 (13)	−0.09704 (10)	−0.08155 (13)	0.0823 (5)	
Cl1	0.34045 (5)	0.02721 (3)	0.65159 (4)	0.07358 (18)	

### Atomic displacement parameters (Å^2^)

**Table:**

	*U*^11^	*U*^22^	*U*^33^	*U*^12^	*U*^13^	*U*^23^
C1	0.0578 (10)	0.0442 (9)	0.0463 (9)	0.0045 (8)	0.0059 (8)	0.0001 (7)
C2	0.1067 (18)	0.0453 (10)	0.0647 (12)	0.0030 (11)	0.0186 (12)	−0.0045 (9)
C3	0.137 (2)	0.0438 (11)	0.0812 (15)	−0.0120 (12)	0.0265 (15)	0.0027 (11)
C4	0.127 (2)	0.0545 (12)	0.0821 (15)	−0.0121 (13)	0.0364 (15)	0.0165 (11)
C5	0.0846 (14)	0.0557 (11)	0.0584 (11)	0.0003 (10)	0.0227 (10)	0.0138 (9)
C6	0.0507 (10)	0.0461 (9)	0.0416 (9)	0.0040 (7)	0.0051 (7)	0.0063 (7)
C7	0.0449 (9)	0.0482 (9)	0.0384 (8)	0.0044 (7)	0.0092 (7)	−0.0006 (7)
C8	0.0390 (8)	0.0452 (8)	0.0417 (8)	0.0003 (7)	0.0105 (7)	−0.0056 (7)
C9	0.0452 (9)	0.0477 (9)	0.0496 (9)	0.0029 (7)	0.0143 (8)	−0.0076 (7)
C10	0.0665 (12)	0.0602 (11)	0.0751 (13)	−0.0034 (9)	0.0417 (10)	−0.0148 (9)
C11	0.0616 (11)	0.0570 (10)	0.0628 (11)	−0.0077 (9)	0.0357 (9)	−0.0076 (9)
C12	0.0406 (9)	0.0476 (9)	0.0516 (9)	−0.0028 (7)	0.0136 (7)	−0.0040 (7)
C13	0.0507 (10)	0.0457 (9)	0.0468 (9)	−0.0013 (7)	0.0190 (8)	−0.0074 (7)
C14	0.0361 (8)	0.0471 (9)	0.0490 (9)	−0.0031 (7)	0.0096 (7)	−0.0035 (7)
C15	0.0394 (9)	0.0471 (9)	0.0455 (9)	−0.0047 (7)	0.0088 (7)	0.0034 (7)
C16	0.0407 (8)	0.0482 (9)	0.0402 (8)	−0.0073 (7)	0.0103 (7)	0.0022 (7)
C17	0.0426 (9)	0.0595 (10)	0.0442 (9)	−0.0054 (8)	0.0071 (7)	0.0054 (8)
C18	0.0496 (10)	0.0785 (13)	0.0425 (9)	−0.0111 (9)	0.0072 (8)	−0.0092 (9)
C19	0.0459 (10)	0.0649 (11)	0.0532 (10)	−0.0096 (8)	0.0201 (8)	−0.0163 (8)
C20	0.0530 (10)	0.0543 (10)	0.0548 (10)	0.0026 (8)	0.0164 (9)	−0.0035 (8)
C21	0.0498 (10)	0.0554 (10)	0.0409 (9)	0.0001 (8)	0.0054 (7)	−0.0026 (7)
C22	0.0970 (18)	0.0700 (14)	0.119 (2)	0.0041 (13)	0.0450 (16)	−0.0304 (14)
C23	0.0385 (9)	0.0451 (8)	0.0451 (9)	−0.0041 (7)	0.0064 (7)	−0.0032 (7)
C24	0.0374 (9)	0.0579 (10)	0.0500 (9)	−0.0025 (7)	0.0068 (7)	−0.0049 (8)
C25	0.0474 (10)	0.0636 (11)	0.0457 (9)	0.0054 (8)	0.0028 (8)	0.0030 (8)
C26	0.0548 (11)	0.0492 (9)	0.0475 (9)	−0.0035 (8)	0.0107 (8)	0.0014 (7)
C27	0.0425 (10)	0.0825 (13)	0.0650 (12)	−0.0124 (9)	0.0093 (9)	0.0169 (10)
C28	0.0406 (10)	0.0816 (13)	0.0578 (11)	−0.0067 (9)	0.0020 (8)	0.0187 (10)
N1	0.0617 (9)	0.0479 (8)	0.0527 (8)	0.0059 (7)	0.0166 (7)	−0.0077 (7)
N2	0.0490 (9)	0.0519 (8)	0.0615 (9)	−0.0005 (7)	0.0056 (7)	−0.0015 (7)
O1	0.1038 (11)	0.0492 (7)	0.0994 (11)	−0.0016 (7)	0.0715 (9)	−0.0109 (7)
O2	0.0469 (7)	0.0501 (7)	0.0803 (9)	0.0050 (5)	0.0088 (6)	−0.0060 (6)
O3	0.0778 (10)	0.0812 (10)	0.0823 (10)	−0.0009 (8)	0.0162 (8)	−0.0380 (8)
Cl1	0.0750 (4)	0.0798 (4)	0.0645 (3)	−0.0019 (3)	0.0190 (3)	0.0220 (3)

### Geometric parameters (Å, º)

**Table:**

C1—N1	1.360 (2)	C14—H14	0.9800
C1—C2	1.411 (3)	C15—N2	1.279 (2)
C1—C6	1.413 (2)	C15—C16	1.462 (2)
C2—C3	1.352 (3)	C16—C21	1.382 (2)
C2—H2	0.9300	C16—C17	1.392 (2)
C3—C4	1.395 (3)	C17—C18	1.367 (3)
C3—H3	0.9300	C17—H17	0.9300
C4—C5	1.353 (3)	C18—C19	1.383 (3)
C4—H4	0.9300	C18—H18	0.9300
C5—C6	1.410 (3)	C19—O3	1.357 (2)
C5—H5	0.9300	C19—C20	1.374 (3)
C6—C7	1.396 (2)	C20—C21	1.381 (2)
C7—C8	1.364 (2)	C20—H20	0.9300
C7—H7	0.9300	C21—H21	0.9300
C8—C9	1.424 (2)	C22—O3	1.416 (3)
C8—C13	1.480 (2)	C22—H22A	0.9600
C9—N1	1.317 (2)	C22—H22B	0.9600
C9—C10	1.491 (3)	C22—H22C	0.9600
C10—C11	1.521 (3)	C23—C28	1.379 (2)
C10—H10A	0.9700	C23—C24	1.381 (2)
C10—H10B	0.9700	C24—C25	1.379 (2)
C11—C12	1.514 (2)	C24—H24	0.9300
C11—H11A	0.9700	C25—C26	1.365 (3)
C11—H11B	0.9700	C25—H25	0.9300
C12—O2	1.460 (2)	C26—C27	1.368 (3)
C12—C13	1.531 (2)	C26—Cl1	1.7396 (18)
C12—C14	1.535 (2)	C27—C28	1.376 (3)
C13—O1	1.2053 (19)	C27—H27	0.9300
C14—C15	1.512 (2)	C28—H28	0.9300
C14—C23	1.513 (2)	N2—O2	1.4210 (18)
			
N1—C1—C2	118.29 (17)	C23—C14—H14	109.4
N1—C1—C6	122.83 (15)	C12—C14—H14	109.4
C2—C1—C6	118.88 (18)	N2—C15—C16	121.43 (14)
C3—C2—C1	120.0 (2)	N2—C15—C14	114.02 (14)
C3—C2—H2	120.0	C16—C15—C14	124.54 (14)
C1—C2—H2	120.0	C21—C16—C17	117.51 (15)
C2—C3—C4	121.1 (2)	C21—C16—C15	121.08 (14)
C2—C3—H3	119.4	C17—C16—C15	121.40 (15)
C4—C3—H3	119.4	C18—C17—C16	120.71 (17)
C5—C4—C3	120.7 (2)	C18—C17—H17	119.6
C5—C4—H4	119.6	C16—C17—H17	119.6
C3—C4—H4	119.6	C17—C18—C19	120.95 (16)
C4—C5—C6	119.9 (2)	C17—C18—H18	119.5
C4—C5—H5	120.0	C19—C18—H18	119.5
C6—C5—H5	120.0	O3—C19—C20	125.29 (18)
C7—C6—C5	123.44 (17)	O3—C19—C18	115.38 (16)
C7—C6—C1	117.14 (15)	C20—C19—C18	119.33 (16)
C5—C6—C1	119.35 (16)	C19—C20—C21	119.37 (17)
C8—C7—C6	120.19 (15)	C19—C20—H20	120.3
C8—C7—H7	119.9	C21—C20—H20	120.3
C6—C7—H7	119.9	C20—C21—C16	122.09 (16)
C7—C8—C9	118.93 (15)	C20—C21—H21	119.0
C7—C8—C13	119.96 (14)	C16—C21—H21	119.0
C9—C8—C13	120.80 (15)	O3—C22—H22A	109.5
N1—C9—C8	122.28 (16)	O3—C22—H22B	109.5
N1—C9—C10	117.66 (15)	H22A—C22—H22B	109.5
C8—C9—C10	120.06 (15)	O3—C22—H22C	109.5
C9—C10—C11	113.61 (15)	H22A—C22—H22C	109.5
C9—C10—H10A	108.8	H22B—C22—H22C	109.5
C11—C10—H10A	108.8	C28—C23—C24	118.38 (16)
C9—C10—H10B	108.8	C28—C23—C14	120.92 (14)
C11—C10—H10B	108.8	C24—C23—C14	120.70 (14)
H10A—C10—H10B	107.7	C25—C24—C23	120.90 (16)
C12—C11—C10	112.05 (15)	C25—C24—H24	119.6
C12—C11—H11A	109.2	C23—C24—H24	119.6
C10—C11—H11A	109.2	C26—C25—C24	119.44 (16)
C12—C11—H11B	109.2	C26—C25—H25	120.3
C10—C11—H11B	109.2	C24—C25—H25	120.3
H11A—C11—H11B	107.9	C25—C26—C27	120.84 (17)
O2—C12—C11	108.75 (14)	C25—C26—Cl1	120.02 (14)
O2—C12—C13	103.51 (13)	C27—C26—Cl1	119.15 (14)
C11—C12—C13	110.57 (13)	C26—C27—C28	119.46 (17)
O2—C12—C14	104.06 (12)	C26—C27—H27	120.3
C11—C12—C14	117.92 (14)	C28—C27—H27	120.3
C13—C12—C14	110.82 (13)	C27—C28—C23	120.98 (16)
O1—C13—C8	121.04 (15)	C27—C28—H28	119.5
O1—C13—C12	121.54 (15)	C23—C28—H28	119.5
C8—C13—C12	117.42 (14)	C9—N1—C1	118.51 (15)
C15—C14—C23	112.46 (13)	C15—N2—O2	109.16 (13)
C15—C14—C12	99.30 (13)	N2—O2—C12	108.05 (11)
C23—C14—C12	116.44 (13)	C19—O3—C22	117.80 (17)
C15—C14—H14	109.4		
			
N1—C1—C2—C3	179.6 (2)	C23—C14—C15—C16	−69.6 (2)
C6—C1—C2—C3	−0.3 (3)	C12—C14—C15—C16	166.66 (15)
C1—C2—C3—C4	0.6 (4)	N2—C15—C16—C21	−174.46 (16)
C2—C3—C4—C5	−0.3 (4)	C14—C15—C16—C21	4.1 (2)
C3—C4—C5—C6	−0.4 (4)	N2—C15—C16—C17	7.0 (2)
C4—C5—C6—C7	−176.28 (19)	C14—C15—C16—C17	−174.36 (15)
C4—C5—C6—C1	0.6 (3)	C21—C16—C17—C18	−0.1 (2)
N1—C1—C6—C7	−3.1 (2)	C15—C16—C17—C18	178.42 (16)
C2—C1—C6—C7	176.80 (17)	C16—C17—C18—C19	1.7 (3)
N1—C1—C6—C5	179.79 (17)	C17—C18—C19—O3	177.97 (17)
C2—C1—C6—C5	−0.3 (3)	C17—C18—C19—C20	−2.0 (3)
C5—C6—C7—C8	178.18 (16)	O3—C19—C20—C21	−179.31 (17)
C1—C6—C7—C8	1.2 (2)	C18—C19—C20—C21	0.7 (3)
C6—C7—C8—C9	1.8 (2)	C19—C20—C21—C16	0.9 (3)
C6—C7—C8—C13	−171.81 (15)	C17—C16—C21—C20	−1.2 (3)
C7—C8—C9—N1	−3.4 (2)	C15—C16—C21—C20	−179.77 (16)
C13—C8—C9—N1	170.19 (16)	C15—C14—C23—C28	−36.3 (2)
C7—C8—C9—C10	176.47 (16)	C12—C14—C23—C28	77.3 (2)
C13—C8—C9—C10	−10.0 (2)	C15—C14—C23—C24	143.45 (15)
N1—C9—C10—C11	−152.60 (17)	C12—C14—C23—C24	−102.91 (18)
C8—C9—C10—C11	27.5 (3)	C28—C23—C24—C25	1.2 (3)
C9—C10—C11—C12	−50.9 (2)	C14—C23—C24—C25	−178.64 (15)
C10—C11—C12—O2	−57.63 (19)	C23—C24—C25—C26	−0.6 (3)
C10—C11—C12—C13	55.4 (2)	C24—C25—C26—C27	−0.4 (3)
C10—C11—C12—C14	−175.68 (14)	C24—C25—C26—Cl1	179.40 (13)
C7—C8—C13—O1	8.9 (3)	C25—C26—C27—C28	0.9 (3)
C9—C8—C13—O1	−164.63 (17)	Cl1—C26—C27—C28	−178.95 (16)
C7—C8—C13—C12	−171.04 (14)	C26—C27—C28—C23	−0.3 (3)
C9—C8—C13—C12	15.5 (2)	C24—C23—C28—C27	−0.7 (3)
O2—C12—C13—O1	−101.47 (19)	C14—C23—C28—C27	179.10 (18)
C11—C12—C13—O1	142.22 (18)	C8—C9—N1—C1	1.6 (3)
C14—C12—C13—O1	9.6 (2)	C10—C9—N1—C1	−178.27 (16)
O2—C12—C13—C8	78.44 (16)	C2—C1—N1—C9	−178.21 (17)
C11—C12—C13—C8	−37.9 (2)	C6—C1—N1—C9	1.7 (3)
C14—C12—C13—C8	−170.53 (14)	C16—C15—N2—O2	179.92 (13)
O2—C12—C14—C15	21.30 (15)	C14—C15—N2—O2	1.2 (2)
C11—C12—C14—C15	141.81 (15)	C15—N2—O2—C12	14.07 (18)
C13—C12—C14—C15	−89.36 (15)	C11—C12—O2—N2	−149.12 (13)
O2—C12—C14—C23	−99.62 (15)	C13—C12—O2—N2	93.29 (14)
C11—C12—C14—C23	20.9 (2)	C14—C12—O2—N2	−22.63 (16)
C13—C12—C14—C23	149.71 (14)	C20—C19—O3—C22	7.0 (3)
C23—C14—C15—N2	109.13 (17)	C18—C19—O3—C22	−172.97 (19)
C12—C14—C15—N2	−14.64 (18)		

### Hydrogen-bond geometry (Å, º)

**Table:**

*D*—H···*A*	*D*—H	H···*A*	*D*···*A*	*D*—H···*A*
C22—H22*B*···O1^i^	0.96	2.54	3.351 (2)	142

Symmetry code: (i) −*x*+1, −*y*, −*z*.
